# Dr. Orin Todd

**Published:** 1896-07

**Authors:** 


### Obituary.

Dr. Orin Todd, of Eminence Co., Ky., died Monday, May 4, 1896, at the Norton Infirmary, Louisville, aged 55 years. He practised medicine for nearly thirty years, and was well known throughout Kentucky. He had never married, but gave the devotion of his life to his profession.

Little hopes of his recovery were entertained from the time of his entrance into the Infirmary, and his numerous friends were not unprepared for the fatal termination. He was buried at Eminence, Ky., by the Odd Fellows.

His death was due to a pernicious form of pneumonia.

